# The next generation of protein super‐fibres: robust recombinant production and recovery of hagfish intermediate filament proteins with fibre spinning and mechanical–structural characterizations

**DOI:** 10.1111/1751-7915.13869

**Published:** 2021-06-30

**Authors:** Paula E. Oliveira, Dong Chen, Brianne E. Bell, Thomas I. Harris, Caleb Walker, Haixia Zhang, Brittany Grob, Randolph V. Lewis, Justin A. Jones

**Affiliations:** ^1^ Department of Biology Utah State University Logan UT 84322 USA; ^2^ Department of Biological Engineering Utah State University Logan UT 84322 USA; ^3^ Key Laboratory of Biotechnology and Bioengineering of State Ethnic Affairs Commission Biomedical Research Center Northwest Minzu University Lanzhou, Gansu China

## Abstract

Native hagfish intermediate filament proteins have impressive mechanical properties. However, using these native fibres for any application is impractical, necessitating their recombinant production. In the only literature report on the proteins (denoted α and ɣ), heterologous expression levels, using *E. coli*, were low and no attempts were made to optimize expression, explore wet‐spinning, or spin the two proteins individually into fibres. Reported here is the high production (~8 g l^−1^ of dry protein) of the hagfish intermediate filament proteins, with yields orders of magnitude higher (325–1000×) than previous reports. The proteins were spun into fibres individually and in their native‐like 1:1 ratio. For all fibres, the hallmark α‐helix to β‐sheet conversion occurred after draw‐processing. The native‐like 1:1 ratio fibres achieved the highest average tensile strength in this study at nearly 200 MPa with an elastic modulus of 5.7 GPa, representing the highest tensile strength reported for these proteins without chemical cross‐linking. Interestingly, the recombinant α protein achieved nearly the same mechanical properties when spun as a homopolymeric fibre. These results suggest that varying the two protein ratios beyond the natural 1:1 ratio will allow a high degree of tunability. With robust heterologous expression and purification established, optimizing fibre spinning will be accelerated compared to difficult to produce proteins such as spider silks.

## Graphical abstract

Robust fibres from recombinant hagfish intermediate filament proteins.

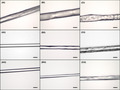

## Introduction

Hagfish are remarkable creatures, not only because of their unique anatomical features but, perhaps most interestingly, their unique adaptation to predation. Hagfish are capable of producing a slime that is reinforced by fibrous proteins. When the slime is expelled, it rapidly expands and can clog the gills of a predator, forcing them to release the hagfish (Fernholm, 1981). While the slime is of keen interest, the reinforcing fibres (intermediate filaments) are also exciting due to their remarkable mechanical properties (Fudge *et al*., 2010). When these fibres are isolated from the slime matrix, draw‐processed, and dried they exhibit mechanical properties similar to those of natural dragline silk from orb‐weaving spiders (Gosline *et al*., 1986; Stauffer *et al*., 1994). The hagfish fibrous thread is heteropolymeric and composed of two proteins, denoted as α and γ (Spitzer *et al*., 1984; Koch *et al*., 1994; Koch *et al*., 1995). These proteins share a common structural architecture: an α‐helical rod domain and N‐ and C‐termini that are not as predominantly α‐helical. In the native fibre, these two proteins coil around each other in a classic coiled‐coil conformation, and when these fibres are draw‐processed the α‐helices convert to β‐sheets, which confer the remarkable mechanical properties (Fudge *et al*., 2003).

In past studies, native hagfish intermediate filament fibres have been dissolved using formic acid and then spun into fibres in an attempt to understand fibre creation and development from spinning systems that are not mimetic to the natural system (Negishi *et al*., 2012). While the mechanical properties of the fibres fell short of their naturally created counterparts, this past study indicated the potential of these two proteins to assemble and form a fibre using alternative spinning technologies. This initial research led to attempts to generate the two proteins in *E. coli*, where the proteins were produced as full‐length natural analogs (Fu *et al*., 2015). When the proteins were purified, the authors demonstrated that they would self‐assemble at the surface of an electrolyte buffer in a native‐like α‐helical conformation from which fibres could be pulled. Furthermore, when the fibres were draw‐processed, the hallmark α‐helix to β‐sheet conversion was observed. Additional characterization with X‐ray diffraction confirmed that the newly formed β‐sheet crystallites were orientated along the axis of the fibre, creating the natural structural elements that infer strength to protein‐based fibres (Fu *et al*., 2017). Again, the mechanical properties fell short of characterized natural fibres. However, this prior study clearly demonstrated the potential of hagfish intermediate filament proteins if the proteins can be efficiently produced, and a spinning method can be developed that generates mechanical properties closer to natural hagfish fibres.

There are intrinsic traits of hagfish intermediate filament proteins that could be beneficial for the efficient production of recombinant fibrous proteins. Hagfish intermediate filament proteins are much smaller (≈ 65 kDa) than the highly studied, and challenging to produce, spider silk proteins (> 300 kDa). They also have a more even distribution of amino acids without the heavy reliance on glycine and alanine found in spider silk. This combination makes them a more amenable target for heterologous expression. Although there are two reports in the literature of recombinant expression of the two hagfish fibrous proteins (Fu *et al*., 2015, 2017), no attempts have been made at optimizing protein production and output using efficient bioreactors. Efficient protein production in bioreactors, and scaling‐up, is mandatory for understanding the potential of these two proteins for incorporation into engineering applications. Additionally, hexafluoroisopropanol (HFIP) has not been explored as a solvent from which to spin hagfish intermediate filament fibres and is known to drive α‐helical conformations in proteins, recreating the natural structures of these proteins (Hirota *et al*., 1997; Maiti *et al*., 2004). While HFIP is not an ideal solvent for commercial or industrial applications, it is apparent that it can easily aid in producing superior fibre mechanical properties to determine the functional capacity of proteins in question.

Supporting this assertion is a recent study that utilized HFIP as a solvent to generate native‐like mechanical properties of spider dragline silk from a full‐length analog of MaSp1 (one of the two proteins that comprise dragline silk) for the first time (Bowen *et al*., 2018). Smaller fragments of MaSp1 were expressed and then the full‐length analog was assembled using the intein system. This experiment illustrates that the recombinant protein forms should be as native‐like as possible with the native structural elements present and that the natural spinning process does not have to be precisely recreated. Rather, HFIP is capable of producing fibres that mimic the mechanical properties of natural fibres.

Recombinant production also allows the unique opportunity to study these two hagfish proteins individually and gain insight into their fibre‐forming abilities, structures, and mechanical properties. There are intermediate filament proteins that are homopolymeric, with one of the most studied being vimentin. In one study, the recombinant form of human vimentin was produced in *E. coli*, purified, and then spun into fibres (Pinto *et al*., 2014). When the vimentin filaments were allowed to self‐assemble, fibres were able to be pulled from the resulting gelatinous film and draw‐processed. These vimentin fibres exhibited maximum tensile strengths of ~ 173 MPa. In the only literature report of fibres spun from recombinantly produced hagfish α and ɣ proteins, at their native ratio of 1:1, using a nearly identical fibre spinning process to the vimentin fibres, the tensile strength reported was 150 MPa (Fu *et al*., 2017).

Further indication that natural arrangements and processes may not need to be exactly replicated to obtain the robust natural properties is the intein spider silk example. The authors created only one of the two proteins of dragline silk and were still able to generate fibre properties that were very close to the native spider silk fibres (Bowen *et al*., 2018). Therefore, this study sought to understand if the individual recombinant forms of the hagfish α and ɣ proteins would assemble into a fibre and to also gain an initial glimpse of their structural elements, mechanical properties, and the interplay between them.

This work demonstrates that very high expression levels of recombinant hagfish intermediate filament (rHIF) proteins are obtainable in *E. coli* using small (~1 l) bioreactors. As a proof of concept, the process was scaled in *E. coli* to the 100 l bioreactor level, which demonstrated similarly high expression. An efficient purification procedure, which is both scalable and economical, is also reported. Finally, fibres that are double draw‐processed, from both the individual proteins and in a native‐like 1:1 combination, were produced, and their resultant mechanical properties and the structural elements of the fibres are reported.

## Results

### Production and Recovery of Recombinant Hagfish Intermediate Filament (rHIF) proteins

**Fig. 1:**
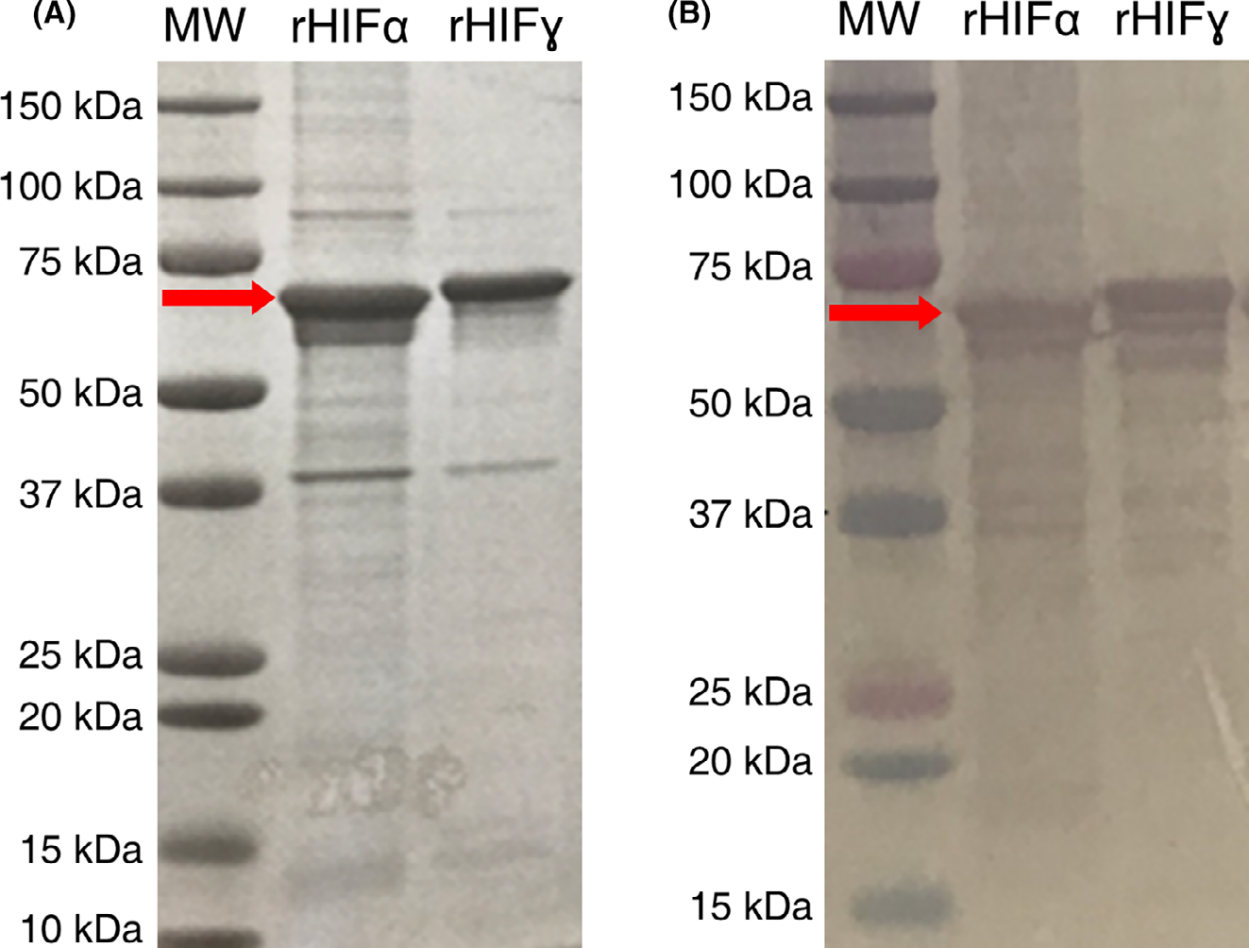
Analytical protein gels of purified protein products running at the expected sized (as indicated by the red arrows). A. Coomassie‐stained SDS‐PAGE of both rHIFα and rHIFɣ_(C387S)_. B. Western blot using anti‐histidine‐tag antibody.

Proteins were purified based upon a standard approach to inclusion body purification in a technique that was not dissimilar from a previous report (Fu *et al*., 2015). As shown in Fig. 1, both proteins were purified to an appreciable degree. In both the Coomassie and western blot analysis, rHIFα migrated further into the gel than rHIFɣ_(C387S)_ even though rHIFɣ_(C387S)_ has a lower molecular weight, likely due to structural differences between the two proteins. Both expression plasmids were verified via DNA sequencing prior to these expression studies (results not shown).

ImageJ analysis of the Coomassie‐stained gel indicates that the proteins are at least 70% pure. Western blot analysis indicates that the majority of the protein is expressed as the full‐length protein. However, banding is apparent below the primary band (premature termination of protein synthesis), which likely biased the ImageJ analysis lower when using the Coomassie‐stained gel for the analysis. Regardless, this level of purity was high enough that the formation and testing of fibres were possible.

Both proteins were successfully synthesized at high levels at a laboratory scale (BioFlo115 at ~ 1 l). At the BioFlo115 level of production, both rHIFα and rHIFɣ_(C387S)_ proteins were produced and recovered on average at ≥ 45 g kg^−1^ cell mass (≥ 8 g l^−1^, Table 1). There was some variability between runs, as reported in the table, possibly due to minor differences in the OD_600_ values at the start of induction, the final OD_600_ values obtained, or the recovery and processing operations. Additionally, rHIFα was induced for 5 h for two runs rather than the standard 4 h induction used for the others. This was an attempt to drive protein expression higher for rHIFα, which was successful. No manipulations were made to the induction time for rHIFɣ_(C387S)_.

**Table 1:** BioFlo115 protein yields for rHIFα and rHIFɣ_(C387S)_ proteins.

Run #	Recovered protein (g)	Volumetric yield (g l^−1^)	Mass yield (g kg^−1^)	Induction time (h)
rHIFα				
1	8	6.7	37.2	4
2	10	8.3	46.5	5
3	12	10.0	55.8	5
Average	10 ± 2.0	8.3 ± 1.7	46.5 ± 9.3	4.7
rHIFɣ_(C387S)_				
1	12	10.0	55.8	4
2	9	7.5	41.9	4
3	8	6.7	37.2	4
Average	9.7 ± 2.1	8.1 ± 1.7	45.0 ± 9.7	4

When production of the two proteins was scaled to the BioFlo610 bioreactor (~ 100 l), the production yields, both per cell mass and per volume, remained relatively consistent with those observed in the BioFlo115 bioreactors (~ 1 l). The production and subsequent recovery of the two proteins resulted in a mass yield of 39 g kg^−1^ cell mass (7.8 g l^−1^) for rHIFα and 45 g kg^−1^ cell mass (8.5 g l^−1^) for rHIFɣ_(C387S)_ (Table S1). As both rHIFα and rHIFɣ_(C387S)_ were expressed as inclusion bodies, no attempts were made to quantify the protein in the soluble fraction. It is important to note that while the BioFlo115 bioreactors were run in triplicate for each construct to validate protein production, the BioFlo610 (~ 100 l) bioreactor was only run once for each construct due to cost and time considerations.

### Mechanical and structural analysis of rHIF fibres

**Fig. 2:**
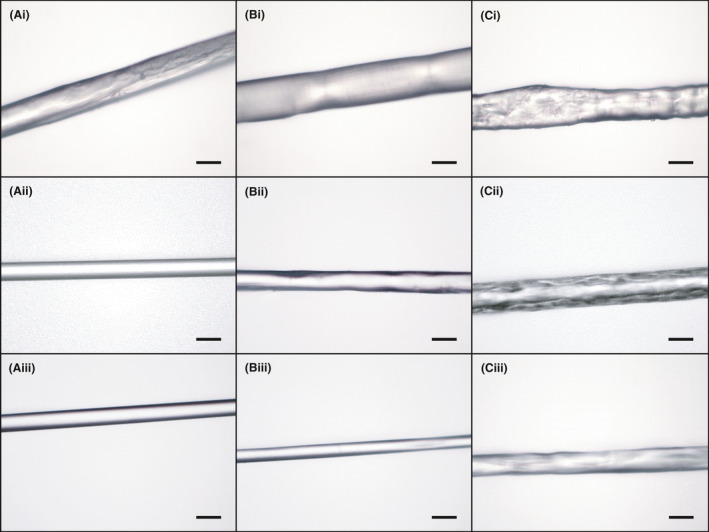
Light microscopy images of fibre at 400X magnification. A. rHIFα fibres, (i) as‐spun, (ii) 1X1X stretch, and (iii) 2X2X stretch. B. 1:1 rHIFα:rHIFɣ_(C387S)_ fibres, (i) as‐spun, (ii) 1X1X stretch, and (iii) 1.5X1.5X stretch. C. rHIFɣ_(C387S)_ fibres, (i) as‐spun, (ii) 1X1X stretch, and (iii) 1.5X1.5X stretch. Scale bars = 30 µm. Fibre images in the bottom row are of stretches that had the smallest average diameters.

Both proteins and the 1:1 rHIFα:rHIFɣ_(C387S)_ combination readily spun fibres from HFIP using isopropyl alcohol (IPA) as the coagulant solution. Due to the brittleness of the as‐spun fibres when dried, mechanical properties were not obtained. Images of the fibres using light microscopy at 400× magnification are presented in Fig. 2. As can be seen, fibres from rHIFα and the 1:1 combination fibres were generally smoother with less surface topography than rHIFɣ_(C387S)_. The rough and inconsistent nature of the rHIFɣ_(C387S)_ fibres could have presented as defects, or weak points, which may have contributed to the mechanical underperformance when compared to the rHIFα and 1:1 rHIFα:rHIFɣ_(C387S)_ protein formulations. However, most of the fibres generally appear smooth on the surface and had a reasonably well‐defined circular cross‐section. The fibre images in the bottom row of Fig. 2 correspond to the stretch at which that protein, or combination, had the highest tensile strength. Fibre images in the top and middle rows are as‐spun and 1X1X stretch, (where no deliberate stretch is applied), respectively, for comparison.

**Fig. 3:**
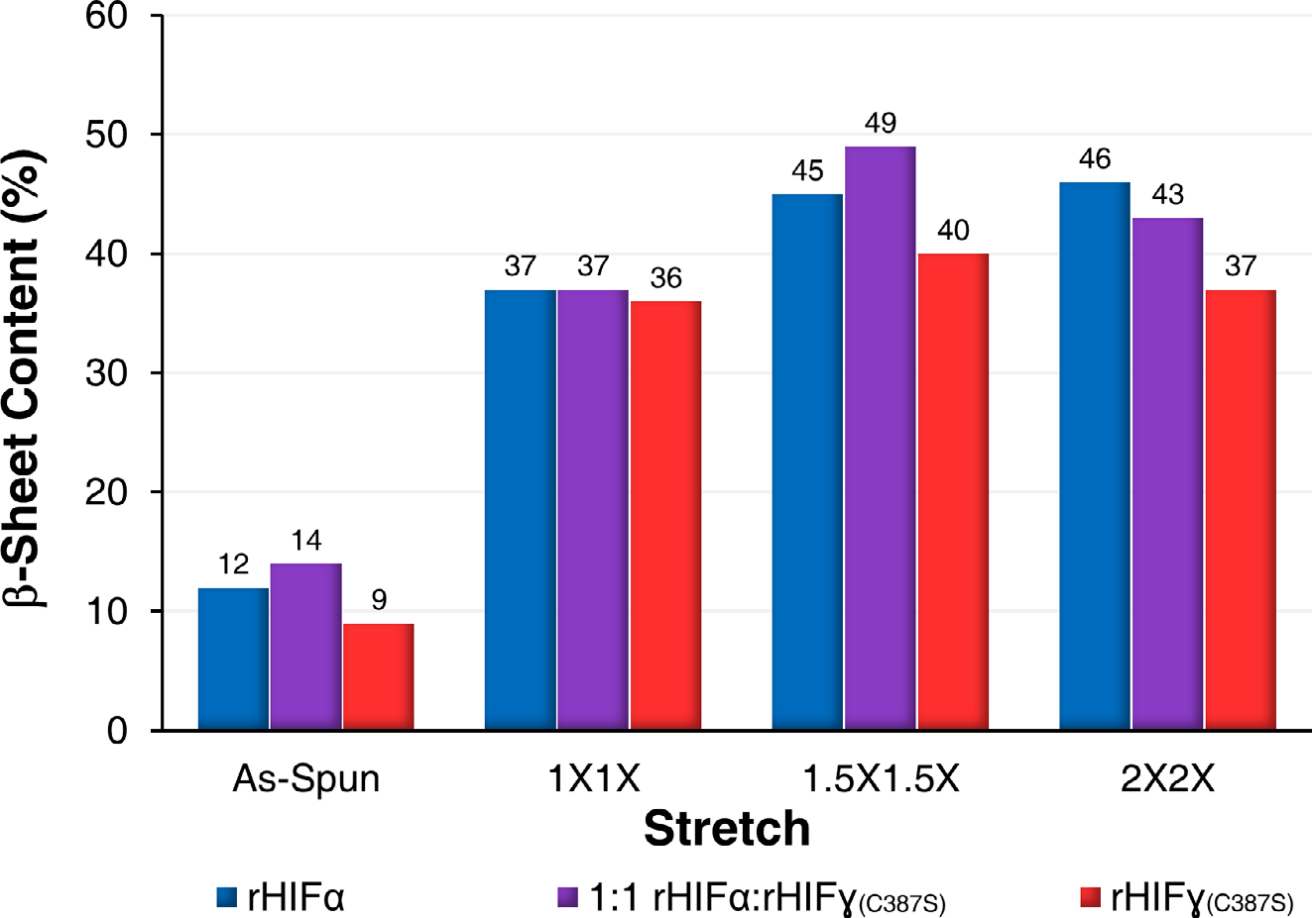
β‐sheet content of the individual protein fibres and 1:1 combination fibres across all of the performed stretches as determined by FTIR deconvolution. Spectroscopic readings were obtained from bundles of at least 50 individual fibres.

The resulting mechanical properties from this study exhibited substantial tensile strength, strain, toughness, and elastic modulus (Table 2). Between the homopolymeric fibres, rHIFα fibres demonstrated better mechanical properties than rHIFɣ_(C387S)_. As shown in Table 2 and Fig. S1, rHIFα did not have any significant changes in the characterized mechanical properties between 1X1X and 1.5X1.5X. There was, however, an 8% increase in β‐sheet content with the additional stretching from 1X1X to 1.5X1.5X (Table 2 and Fig. 3). At both stretches, rHIFα displayed the highest recorded strains at 0.92 and 0.87 mm mm^−1^, and appreciable tensile strength at 104 and 106 MPa, respectively. This combination of the highest strains and higher strengths resulted in the rHIFα fibres having the highest toughness of all fibres tested with average values of 72 and 73 MJ m^‐3^, for 1X1X and 1.5X1.5X, respectively. A substantial decrease in fibre diameter and significant changes for all mechanical properties (Fig. S1) were observed at the 2X2X stretch without additional β‐sheet recruitment. The fibre diameter decreased nearly 30% (16.7 µm), tensile stress increased 60% (169 MPa), strain decreased by 73% (0.23 mm mm^−1^), toughness decreased by 55% (33 MJ m^‐3^), and the elastic modulus improved by 49% (5.4 GPa). It is notable that while having one of the highest tensile strengths and elastic modulus, the reduction in strain resulted in these fibres having the third lowest average toughness of all fibres. When these mechanical properties and the fibre diameter are compared to the 1:1 rHIFα:rHIFɣ_(C387S)_ combination fibres, at 1.5X1.5X, it is evident that the mechanical properties of rHIFα are remarkably similar with only the tensile strength being statistically significantly different (Fig. S1).

**Table 2:** Measured mechanical properties and structural compositions of spun rHIF fibres.

Protein(s)	*n*	Mechanical properties				Structural composition		
		Diameter (µm)	Tensile strength (MPa)	Strain (mm mm^−1^)	Toughness (MJ m^‐3^)	Elastic modulus (GPa)	β‐Sheets (%)	α‐Helices/Random Coils/Turns (%)
As‐Spun								
rHIFα	–	–	–	–	–	–	12	88
1:1 rHIFα:rHIFγ_(C387S)_	–	–	–	–	–	–	14	86
rHIFγ_(C387S)_	–	–	–	–	–	–	9	91
1X1X								
rHIFα	20	23.4 ± 2.2	104 ± 22	0.92 ± 0.31	72 ± 22	3.4 ± 0.7	37	63
1:1 rHIFα:rHIFγ_(C387S)_	20	25.7 ± 1.3	84 ± 11	0.42 ± 0.26	28 ± 17	3.2 ± 0.5	37	63
rHIFγ_(C387S)_	25	36.4 ± 8.3	74 ± 14	0.04 ± 0.01	1.8 ± 0.6	3 ± 0.5	36	64
1.5X1.5X								
rHIFα	23	23.8 ± 0.6	106 ± 15	0.87 ± 0.18	73 ± 22	3.6 ± 0.3	45	55
1:1 rHIFα:rHIFγ_(C387S)_	25	16.2 ± 0.8	199 ± 24	0.21 ± 0.07	35 ± 14	5.7 ± 0.9	49	51
rHIFγ_(C387S)_	25	20.2 ± 1.4	122 ± 23	0.62 ± 0.11	61 ± 13	3.5 ± 0.7	40	60
2X2X								
rHIFα	23	16.8 ± 0.8	169 ± 17	0.23 ± 0.04	33 ± 7	5.4 ± 0.8	46	54
1:1 rHIFα:rHIFγ_(C387S)_	25	17.7 ± 1.2	149 ± 26	0.56 ± 0.11	66 ± 18	4.1 ± 0.6	43	57
rHIFγ_(C387S)_	24	20.3 ± 3.0	120 ± 25	0.47 ± 0.10	43 ± 8	3.1 ± 0.6	37	63

At the 1X1X stretch factor for the1:1 rHIFα:rHIFɣ_(C387S)_ combination fibres produced fibres that were not as robust as the rHIFα fibres but were mechanically superior to the rHIFɣ_(C387S)_ fibres. In fact, the mechanical properties of the 1:1 combination fibres fell almost perfectly between the values of the rHIFα and the rHIFɣ_(C387S)_ samples (Table 2). The 1:1 combination fibres at the 1.5X1.5X stretch demonstrated the highest tensile strength (199 MPa), the smallest fibre diameter (16.22 µm), the lowest strain (0.21 mm mm^−1^), and the highest elastic modulus (5.7 GPa), as well as the highest β‐sheet content (49%) of all characterized fibres (Fig. 3). This stretch also yielded the greatest increase, of 12%, for the β‐sheet content of all stretched fibres. The 1.5X1.5X stretch appears to be optimal for obtaining maximum tensile stress and elastic modulus for this particular protein formulation. However, the lower strain resulted in a lower toughness (35 MJ m^‐3^). Interestingly, when the 1:1 combination fibres were stretched at 2X2X, there were significant differences across all mechanical properties (Fig. S1). The tensile strength decreased by 29% (149 MPa), the strain more than doubled (0.56 mm mm^−1^), which nearly doubled the average toughness (66 MJ m^‐3^), and the elastic modulus was also 33% lower (4.1 MPa). However, even with those reductions, this combination resulted in the third highest tensile strength of all the fibre combinations, demonstrating a relatively high degree of tunability through manipulations of draw‐processing.

The rHIFɣ_(C387S)_ fibres did not achieve a maximum value for any mechanical property. The 1X1X rHIFɣ_(C387S)_ consistently presented with the lowest mechanical properties for all of the characterized fibres (Table 2). The rHIFɣ_(C387S)_ fibres appeared to be optimally stretched at around 1.5X1.5X as the fibre diameter decreased by 57% (20.2 µm) and the highest tensile strength and strain of all rHIFɣ_(C387S)_ fibres (122 MPa and 0.62 mm mm^−1^) were obtained, which resulted in an appreciable toughness (61 MJ m^‐3^). Additional stretching to 2X2X resulted in the reduction of all measured mechanical properties with significant decreases occurring for all properties with the exception of the elastic modulus (Fig. S1). The β‐sheet content for rHIFɣ_(C387S)_ fibres at 2X2X also decreased to nearly 1X1X stretch levels when draw‐processed further. All rHIFɣ_(C387S)_ fibres consistently demonstrated the lowest β‐sheet content (Fig. 3 and Table 2) and the most limited range of elastic modulus (3–3.5 GPa).

All fibres saw substantially increased β‐sheet content from the as‐spun to 1X1X (Fig. 3 and Table 2), which was simply a result of the fibre being threaded through the instrument and exposed to the stretch bath solutions. As‐spun fibres ranged from 9−14% β‐sheet content, and all fibres increased nearly identically to 36–37% at 1X1X. Even with this sizeable initial increase, further stretching resulted in additional β‐sheet recruitment, although in smaller increments, which resulted in substantial improvements in mechanical properties. Particularly notable is that when the 1:1 combination fibres were optimally stretched at 1.5X1.5X, the β‐sheet content rose above both rHIFα and rHIFɣ_(C387S)_, which did not happen at any other stretch, although this was observed for the as‐spun fibres that had not been stretched. Additionally, the β‐sheet content of both 1:1 rHIFα:rHIFɣ_(C387S)_ combination and rHIFɣ_(C387S)_ fibres decreased when draw‐processed to the 2X2X level, which correlates with the measured mechanical properties. In contrast, the β‐sheet content and mechanical properties of rHIFα fibres increased at this level.

## Discussion

The protein purification process is based on a standard approach to inclusion body purifications. When expressed as inclusion bodies, the insolubility of both proteins allows the use of urea to remove more soluble contaminating proteins. For this study, the process was accomplished using batch centrifugation. However, inclusion body purifications can be scaled with large volume filtration systems (Forman *et al*., 1990). Large volume filtration systems should also allow for improvements in purity via additional processing steps and, if necessary, refolding of the proteins in a controllable manner. Further refinements of the purification process will predictably reduce the overall yield as more impurities are removed. However, given that this study induced cultures at an OD_600_ of ≈ 60, there is sufficient room to improve cell density, and therefore the total cell mass produced through the optimization of bioreactor media and feedstocks. There are several reports in the literature of *E. coli* in bioreactors achieving optical densities above 100 (Li and Sha, 2017). If cell density correlates in a linear manner with total protein production and recovery, then yields in excess of 13 g l^−1^ could be expected.

A previous report on recombinantly produced hagfish intermediate filament proteins α and ɣ only achieved yields of 0.01–0.02 g l^−1^ when using *E. coli* as a host (Fu *et al*., 2015). Using methodologies developed during this study, substantial improvements were made to the protein yields, which were increased by 325‐ to 1000‐fold from previous reports. An efficient and scalable purification process also allowed for the production of rHIF fibres with the highest mechanical properties yet reported for recombinant hagfish intermediate filament proteins. Furthermore, these fibres were produced with conventional wet‐spinning techniques, instead of solution casting and drawing, which allowed for continuous fibre lengths to be produced that could be draw‐processed as desired.

Both rHIFα and rHIFɣ_(C387S)_ were produced and recovered at a laboratory production scale at ≥ 45 g kg^−1^ cell mass (≥ 8 g l^−1^). This process was then scaled to a relatively large 100 l bioreactor, where the proteins were again recovered at similarly high levels. This makes them very economical, according to a recent techno‐economic report on the production of spider silk (Edlund *et al*., 2018). A key bottleneck to the production of recombinant spider silk proteins is a general inability to produce native‐sized proteins, with all structural elements present, at a sustainable level that supports systematization into engineering applications (Xia *et al*., 2010). The two recombinant forms of hagfish intermediate filaments investigated in this study do not suffer from this same problem, possibly due in part to the smaller molecular weights and less repetitive amino acid contents. Not only are they readily produced in *E. coli*, but they are also produced as largely full‐length synthetic analogs of the native proteins. Additionally, as Table 3 demonstrates, the levels of expression and recovery surpass, by more than twofold, any reports of other structural fibre‐forming proteins when produced in *E. coli*.

**Table 3:** Comparative expressions/yields of recombinantly produced fibre‐forming proteins.

Protein(s)	Molecular weight (kDa)	Volumetric expression/Yield (g l^−1^)	References
rHIFα	70	6.5–10	This Work
rHIFɣ_(C387S)_	66	6.7–10	This Work
(rec)EsTKα, (rec)EsTKɣ	66.7, 62.8	0.01–0.02	Fu *et al*. (2015)
MaSp1	100.7, 284.9	0.5–2.7	Xia *et al*. (2010)
ADF3, ADF4	11.9–59.3	0.01–0.36	Huemmerich *et al*. (2004)
MaSp2	201.6	3.6	Yang *et al*. (2016)
Honey Bee Silk	40	2.5	Weisman *et al*. (2010)
Suckerin‐39	39–42	0.0043–0.0202	Ding *et al*. (2014)

In the only other literature report on the spinning of synthetically produced hagfish intermediate filament proteins α and ɣ into fibres, the authors were able to achieve tensile strengths between 25 and 150 MPa when the fibres were draw‐processed and dried (Fu *et al*., 2017). The study used highly purified proteins and a refolding process to attempt to create a natural coiled‐coil structure. The fibre spinning process was performed by hand and utilized the solution casting and drawing procedure, which could only be performed with small volumes (~ 2 µl) of protein dope solutions. Finally, the fibres were also cross‐linked with glutaraldehyde to take advantage of the lysine concentration of the two proteins, and those fibres presented tensile strengths up to 250 MPa with predictably very little strain (Fu *et al*., 2017). Although the fibres presented in this previous study are impressive, the system has several limitations, including the yields, protein assembly methods, and the spinning process.

This study utilizes HFIP as a solvent due to its high propensity to generate α‐helical conformations in proteins, which is the native conformation of the hagfish proteins. It has also been shown to produce protein fibres that recapitulate the natural fibre mechanical properties from recombinant spider silk protein analogs (Xia *et al*., 2010; Bowen *et al*., 2018). When these predominantly α‐helical rHIF proteins are forced out of solution in the coagulation bath, the proteins must interact or a fibre will not form, possibly creating a native‐like coiled‐coil structure. Since the two proteins and the 1:1 rHIFα:rHIFɣ_(C387S)_ combination readily formed fibres and demonstrated a substantial proportion of α‐helical and random structures in the as‐spun fibres, the characteristic coiled‐coil structure presumably formed to some extent, although this was not directly measured in this study.

The substantial recruitment of β‐sheet demonstrated the conversion of α‐helices to β‐sheets from as‐spun to 1X1X (where no deliberate stretch is applied), which supports that the transition occurs even when minimal force is applied to the fibre. The varied compositions of the stretch baths and the observed changes also suggest that environmental conditions can influence this structural transition. Increased stretching, or draw‐processing, was successful in recruiting additional β‐sheets that strongly correlate with the impressive gains in mechanical properties, especially tensile strength and elastic modulus (Table 2).

The highest average tensile strength and elastic modulus values were obtained by the 1:1 rHIFα:rHIFɣ_(C387S)_ combination fibres indicating a synergistic effect of having both proteins present in this system (Table 2 and Fig. 3). The β‐sheet content of the 1:1 combination fibres at the 1.5X1.5X stretch is particularly indicative of this synergy. Here, the β‐sheet content exceeded that of both rHIFα and rHIFɣ_(C387S)_ when spun individually (Table 2 and Fig. 3). At both 1X1X and 2X2X stretches, the 1:1 combination fibres contained a blend of the β‐sheet content of individual rHIFα and rHIFɣ_(C387S)_ fibres. Indicating that when draw‐processed to an optimal degree, more β‐sheets can be recruited when both proteins are present than either protein can achieve individually.

Notably, the rHIFα fibres were nearly as impressive as the 1:1 combination fibres and exceeded the mechanical properties reported from the previous study (Fu *et al*., 2017). The rHIFα fibres demonstrated the broadest range of strain, nearly doubling in initial length for the 1X1X stretch. However, when stretched further to 2X2X, the tensile strength, elastic modulus, strain, and β‐sheet content were similar to the best‐performing fibres in this study; the 1:1 combination at 1.5X1.5X. Unlike the other two protein formulations studied in this investigation, the β‐sheet content of rHIFα fibres increased for all stretches and did not drop at the highest stretch performed. This suggests that each protein may respond uniquely to processing and tuning. Particularly indicative of the tunability observed for these proteins is that between the 1X1X fibres and the 2X2X fibres, rHIFα lost 70% strain and gained nearly 63% more tensile strength.

Mechanical properties for rHIFɣ_(C387S)_ fibres were lower than both rHIFα and the 1:1 rHIFα:rHIFɣ_(C387S)_ combination fibres. However, they are still mechanically impressive when compared to previously reported fibres. Again, the stretch factor of 1.5X1.5X for rHIFɣ_(C387S)_ fibres produced the best fibres for this protein formulation, with some properties exceeding the prior synthetic proteins and regenerated natural slime threads (Negishi *et al*., 2012; Fu *et al*., 2017). Even though all fibres were spun with the same conditions and parameters, the rHIFɣ_(C387S)_ fibres were generally larger in diameter and displayed larger surface deformities than either rHIFα or the 1:1 combination fibres. Both of these factors likely played a role in the observed mechanical properties. One potential explanation of the higher occurrence of these features for the rHIFɣ_(C387S)_ fibres may be that the protein is less organized or does not assemble as efficiently with this spinning method. The removal of the single cysteine from the sequence is not thought to play a role in the lower mechanical performance observed. In the native fibres, it is unlikely to form a disulfide bond and contribute mechanically to the performance of the fibres (Fudge and Gosline, 2004; Fudge *et al*., 2010). However, since both proteins were expressed as inclusion bodies, and the omission of the cysteine from the rHIFɣ sequence was an unsuccessful attempt to produce soluble rHIFɣ, reinserting the cysteine should be explored as another route to obtain specifically tunable fibres.

Although not directly measured in this study, there is suggestive evidence that by stretching the fibres, the β‐sheets are being orientated along the axis of stretch as has been reported for native and recombinant hagfish intermediate filament fibres, spider silks, and others (Lefèvre *et al*., 2007; Negishi *et al*., 2012; Sampath *et al*., 2012; Pinto *et al*., 2014; Fu *et al*., 2017). This is particularly apparent for the rHIFα fibres. At the 1.5X1.5X stretch, the diameters averaged ~ 24 µm with 45% β‐sheet content. When stretched to 2X2X, the diameters of the fibres dropped to ~ 17 µm, and the β‐sheet content increased a negligible amount (45–46%), yet the fibres demonstrated a 63% increase in tensile strength and a 40% improvement in elastic modulus. It is unlikely that the improvements were the result of a 1% increase in β‐sheet content.

For this study, only the individual proteins and their natural 1:1 combination were explored. However, the stark differences between the mechanical properties of the two proteins are intriguing as homopolymeric fibres. Varying the ratio beyond the natural 1:1 ratio will likely allow for the production of fibres with extensive tunability. For instance, producing a fibre with a high ratio of rHIFɣ_(C387S)_ would likely provide a less rigid fibre (lower elastic modulus), while the presence of rHIFα would improve the tensile strength of the fibre due to its increased ability to form β‐sheets. The ratio of the two proteins could be adjusted along with the post‐spin draw to produce a fibre that exactly matches a mechanical requirement.

While this study indicates that these two proteins can be expressed at high levels in *E. coli*, the natural properties of hagfish intermediate filaments were not fully reproduced, although the highest mechanical properties were recorded to date for recombinant hagfish intermediate filament proteins. It should be noted that no attempts to optimize the spinning process were performed, and spinning was solely conducted with the reported parameters. Additionally, both proteins contained a histidine‐tag and no attempts were made to understand the influence of the histidine‐tag on fibre mechanical properties, if any. Neither the expression vector or gene sequences were designed with a proteolytic site to remove the tag.

It is clear that the fibre diameter has a substantial contribution to the lower mechanical properties observed. When isolated from the slime and dried, natural hagfish fibres are reported at 1.27 µm, and when stretched and dried, the diameter decreases to 1.07 µm (Fudge *et al*., 2010). The finest fibres produced from this study were 15 times larger in diameter than their natural counterparts. Reduction in the diameter and thus the cross‐sectional area of the fibres, even by relatively small increments, should allow for substantial improvements in the mechanical properties. Supporting this, as indicated in Table 2, is that as the fibre diameters are decreased through draw‐processing, the tensile strength and elastic modulus increase, similar to other protein‐based fibres. Conversely, as the diameter and strain increase, even if only slightly, there is a corresponding decrease in elastic modulus and tensile strength. Further refinements in the spinning process will likely result in finer fibres with more native‐like mechanical properties. A variety of parameters could be explored when spinning these proteins individually or in combination. Previous works have successfully utilized formic acid to solvate the proteins and may produce fibres with different mechanical properties in this system. The actual amount of protein solvated, or concentrations, could relate to final fibre properties. Physical spinning parameters could also affect fibre mechanical properties such as, needle diameter, coagulation bath extrusion rate, and the speed at which the fibres traverse the system and thus the exposure time to the stretch bath solutions. Additional parameter control could also be achieved by varying the compositions of the stretch baths with alcohols other than isopropyl alcohol, varying the water ratios within the baths, and exploring other bath solutions to further refine the fibre mechanical properties. Alternative spinning techniques that produce very fine fibres, such as electrospinning, should also be explored.

## Conclusion

This work has demonstrated that the production of recombinant hagfish intermediate filament proteins, using *E. coli* as a heterologous host, occurs at high enough levels to be commercially favourable without any additional optimization. Furthermore, the initial scale‐up of the production system did not result in any significant reductions in the expression of the recombinant proteins. An effective purification strategy was also developed for the recovery of the rHIF proteins, which resulted in yields that surpass other similarly produced fibrous structural proteins. The large quantities of recovered protein allowed for rapid progress to investigate fibre production methods and ultimately resulted in noteworthy fibres. The fibres spun from these efforts yielded some of the highest yet reported mechanical properties for these recombinantly produced forms of hagfish intermediate filament proteins. Recombinant production also allowed the opportunity to study these proteins in non‐mimetic states that do not occur naturally to better understand the individual protein functions and roles. Through these individual and combined studies of the proteins and their fibres, it was determined that a broad range of tunability is possible for the mechanical properties of these synthetic fibres, which can be characteristically controlled by the proteins utilized, the protein structures, the processing methods, and possibly other unexplored factors. This study provides the first evidence that recombinant hagfish intermediate filament proteins can be produced at high enough levels to be a viable source of material for high‐performance fibrous protein materials.

## Experimental procedures

### Expression vector construction

**Fig. 4:**
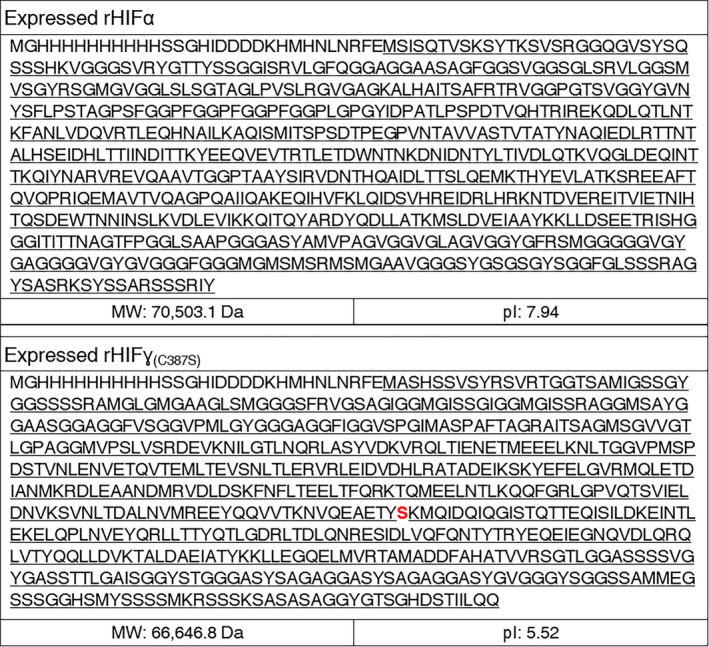
Expressed sequences for both rHIFα (top) and rHIFɣ_(C387S)_ (bottom). The underlined sequences represent the natural sequence. Sequence not underlined represents sequences added as part of the expression vector, including the histidine‐tag sequence. Note: the cysteine at position 387 has been replaced with serine (C387S) and is red in the rHIFɣ_(C387S)_ (bottom) sequence.

The genes encoding hagfish α and ɣ proteins have been previously identified (Koch *et al*., 1994; Koch *et al*., 1995). One change was made in the natural ɣ sequence. As is indicated in Fig. 4, the cysteine that is ordinarily present in the natural hagfish ɣ protein sequence was removed and replaced with serine. The removal of the cysteine was an attempt to improve the purification of the proteins by removing the ability to form disulfide bonds. Provided that both proteins were expressed at high levels as inclusion bodies, this may not have been necessary. Finally, the pET‐19k vector included a 10× histidine‐tag at the N‐terminal. While this tag was included, it was not utilized for purification. Rather, the histidine‐tag was used in western blot analysis to confirm the identity of the produced proteins. The recombinant proteins are denoted as recombinant hagfish intermediate filament alpha (rHIFα) or gamma (rHIFɣ_(C387S)_). The specific amino acid substitution denoted for rHIFɣ_(C387S)_ is identified in the sequence in Fig. 4.

The full‐length gene sequences were codon‐optimized for expression in *E. coli* using ThermoFisher gene Optimizer™ software and were synthesized by ThermoFisher Scientific (Grand Island, NY, USA).

The pET19k cloning vector was generated in our laboratory by modifying the pET19b vector (Novagen, St. Louis, MO, USA) by replacing the ampicillin resistance gene with the kanamycin resistance gene from the pET26b vector (Novagen) Fig. S2. Synthesized hagfish genes were inserted into the pET19k vector at the restriction sites of NdeI and BamHI. The resulting vectors were transformed into *E. coli* BL21 (DE3) chemically competent cells (New England Biolabs, Ipswich, MA, USA) to produce the two recombinant hagfish proteins.

### Expression of rHIFα and rHIFɣ_(C387S)_ proteins

Before scaling to the BioFlo610 (~ 100 l) level of production, protein expression was validated first in shaker flasks (data not shown) and in New Brunswick Scientific BioFlo115 (~ 1 l) bioreactors. Both rHIFα and rHIFɣ_(C387S)_ were expressed as inclusion bodies at all scales. Each construct was produced in triplicate using the BioFlo115 bioreactors. From each batch, the protein was purified, lyophilized, and weighed. The protocols to scale‐up from the BioFlo115 to BioFlo610 bioreactors remained the same in terms of instrument operation, media formulation, and feeds given that all of the reported fed‐batch fermentations were conducted with New Brunswick bioreactors using the BioCommand software. As such, the protocol and media components are reported only once. The entire cell mass from the BioFlo610 runs was not able to be purified at one time, as was performed with the BioFlo115 runs, due to the relatively large cell masses (19 kg for rHIFα and 13 kg for rHIFɣ_(C387S)_) and equipment limitations. Instead, multiple purifications were performed on the total cell mass from each run. The protein yield was then averaged across those purifications.

The rHIFα and rHIFɣ_(C387S)_ protein expressions were scaled up in a New Brunswick Scientific BioFlo610 bioreactor. The first seed solution was grown in LB medium (100 ml) plus 10 g l^−1^ glucose and 100 mg l^−1^ kanamycin in a 500 ml Erlenmeyer flask with rotary shaking (220 rpm) at 37°C to an OD_600_ of one. The first seed solution was then inoculated into LB (3 l) with 10 g l^−1^ glucose and 100 mg l^−1^ kanamycin in a 10 l bottle to produce the second seed solution. The second seed solution was grown with rotary shaking (130 rpm) at 30°C to an OD_600_ of one. The second seed was grown at a lower temperature to slow growth and increase the time required to reach the desired OD_600_ value, for timing purposes. Inoculum culture was pumped into the BioFlo610 fermenter with 50–70 l of sterilized modified K12 medium (Table S2), 100 mg l^−1^ kanamycin, and 0.02% v/v C‐8840 antifoam (New London Chemical, Lakeland, FL, USA). The starting fermentation temperature was set at 37°C, and the pH setpoint was 6.8, which was regulated by the automatic addition of 20% ammonium hydroxide throughout the fermentation. A dissolved O_2_ level of 80% was cascade controlled by agitation (150–400 rpm), gas flow (10–100 SLPM), and bubbling air and O_2_ (0–100%) into the culture. Glucose feeding solution (24–28 l) was fed during the fermentation controlled by the BioCommand software. The glucose level was monitored by a ReliOn Prime Blood Glucose Monitoring System. Induction of protein expression was initiated when an OD_600_ value of 55–60 was obtained. The target proteins, rHIFα or rHIFɣ_(C387S)_, were induced with 1 mM IPTG at 28°C. After 4 h of induction, when the OD_600_ value was around 100, the culture was harvested by centrifugation for 15 min at 8000–10 000 rcf at 4°C, and the cell pellets were stored in a −80°C freezer until processed for purification.

### Protein purification and verification

#### Cell lysis

The frozen hagfish rHIFα and rHIFɣ_(C387S)_ cell pellets were thawed and resuspended at 10 ml g^−1^ of cells in lysis buffer (50 mM Tris and 200 mM NaCl at pH 7.9 (rHIFα) or pH 5.5 (rHIFɣ_(C387S)_) with 200 µg ml^−1^ lysozyme). The solutions were sonicated for 10 cycles of 10 s, with intervals of 45 s between cycles with a VCX 1500 (Sonics Vibracell, Newton, CT, USA).

#### Inclusion body washing

Lysate was centrifuged at 10 000 rcf for 15 min at 4°C, the resulting pellets (hagfish rHIFα and rHIFɣ_(C387S)_ inclusion bodies) were resuspended in wash buffer 1 (100 mM Tris, 5 mM EDTA, 0.5 M urea, 2% v/v Triton X‐100, and 5 mM DTT; pH 7.9 for rHIFα and pH 5.5 for rHIFɣ_(C387S)_) at 5 ml g^−1^ cells. After centrifugation, the pellets were rewashed with wash buffer 1 and followed by two washes of wash buffer 2 (100 mM Tris, 5 mM EDTA, and 5 mM DTT; pH 7.9 for rHIFα and pH 5.5 for rHIFɣ_(C387S)_) at 5 ml g^−1^ cells all with centrifugation parameters described above. The pellets were then washed with 1:1 1X TAE: isopropyl alcohol (IPA) (1X TAE: 40 mM Tris, 1 mM EDTA, and 20 mM acetic acid). A final set of washes of 1:1 deionized water:IPA were performed until the conductivity of the supernatant was <20 µS cm^−1^. The washed proteins were then lyophilized. Production yields were determined by weighing the recovered dry protein and comparing it to either the cell mass or the final working volume in the bioreactor to give grams of protein recovered per kilogram of cell mass (g kg^−1^) or grams of protein recovered per litre of media (g l^−1^).

#### SDS‐PAGE coomassie analysis

The purified proteins were mixed 1:1 v/v with 2× Laemmli Sample Buffer (Bio‐Rad, Hercules, CA, USA) and heat‐treated at 100°C for 5 min before loading on polyacrylamide gels (Novex 4‐20% Tris‐Glycine from ThermoFisher). A dual‐colour protein standard (Bio‐Rad) was included on all gels. The gels were allowed to run for 60 min at a constant 110 V. After SDS‐PAGE analysis, the gels were rinsed using deionized water before being stained with 5 ml Bio‐Rad Bio‐Safe Coomassie Stain for 60 min. The gel was then destained using deionized water for 60 min. ImageJ (NIH) was then utilized to determine the protein purity by selecting each individual lane, isolating the main protein band, and then subtracting the main protein band from contaminating bands within the same lane.

#### Western blot analysis

The protein samples separated by SDS‐PAGE were transferred to a PVDF/Immobilon™‐P Membrane (Millipore, Burlington, MA, USA) by electroblotting using the Mini Trans‐Blot System (Bio‐Rad). Blots were set up as specified by the manufacturer (Bio‐Rad). All transfers were performed under a constant 200 mA for 50 min. After fixing of the proteins, the membranes were subjected to immunoblotting analyses using 1X TBS‐T (1× TBS‐T: 20 mM Tris, 140 mM sodium chloride, and 0.05% v/v Tween‐20, pH 7.4) and Carnation dehydrated milk as the blocking reagents at a concentration of 5% w/v. The primary antibody was an anti‐6X his epitope tag, (mouse) antibody (Rockland, Limerick, PA, USA) at a 1:5000 dilution, and the secondary was an anti‐mouse IgG (H + L) AP conjugate antibody (Promega) at a 1:10 000 dilution. Each addition of antibody was allowed to mix on the membranes for 30 min, with 15 min rinses of TBS‐T in between each antibody and before the development addition. For the detection of alkaline phosphatase activity on the PVDF membrane, 1‐Step™ NBT/BCIP Substrate Solution (ThermoFisher) was used as specified by the manufacturer.

### Fibre preparation and production

The dried proteins were dissolved in 1,1,1,3,3,3‐hexafluoro‐2‐propanol (HFIP) (Oakwood Chemical, Rochester, MI, USA) at a concentration of 5% w/v. The two protein types were weighed out into a 4 ml capacity glass vial (Wheaton) for a 2 ml dope of 5% w/v concentration, and then 2 ml of HFIP were added. The vial was then capped and sealed with Parafilm. Additionally, a combination dope was made by mixing equal amounts of both, rHIFα and rHIFɣ_(C387S)_ proteins in an overall 5% w/v protein concentration in 2 ml of HFIP. The dopes were designated by their protein content as rHIFα, 1:1 rHIFα:rHIFɣ_(C387S)_ or 1:1 combination, and rHIFɣ_(C387S)_.

Protein dope solutions were allowed to solvate for 7 d. Solvation occurred prior to this, but was permitted additional time to ensure that all proteins were dissolved. The dopes were then centrifuged at 18 000 rcf for 10 min to remove any remaining particulates. The supernatant was loaded into a BD 3 ml syringe. A spinning port was placed onto the luer‐lok end of the syringe. This was accomplished using the necessary PEEK tubing adapters for connecting PEEK tubing (I.D. 0.254 mm) to a syringe (Fig. S3).

The loaded syringe system was placed into a custom extrusion spinning machine, as has been previously described (Fig. S4) (Copeland *et al*., 2015). The dope was extruded into the coagulation bath at a rate of ~25 µl min^−1^ using a plunger press. Spinning bath contents were: coagulation bath of 99% IPA (high‐purity), the first stretch bath at 80% IPA:20% ultra‐pure water, and the final stretch bath solution at 20% IPA:80% ultra‐pure water. The as‐spun fibres were only extruded into the coagulation bath without being threaded through the spinning instrument. The stretches are reported as 1X1X, 1.5X1.5X, and 2X2X. For example, the 2X2X stretch denotes that the fibre was draw‐processed to 2X the initial length in the first stretch bath (80% IPA) and then drawn again to 2X the length in the second stretch bath (20% IPA) for a total of 300% draw‐processed. Stretches are obtained by varying the rotational speed of the godets in the stretch baths; a 2X stretch is obtained when the circumference of second godet covers twice the distance of the first godet. The 1X1X stretch denotes that no deliberate stretch was applied in either bath, or that all godets were rotating at the same speed. The fibre was simply pulled from the coagulation bath, threaded through the instrument, and collected on the winding spool. To promote drying and prevent fibres from sticking together or sticking to the collecting spool, drying lamps (DL in Fig. S4) were utilized for each spin between the final godet and the spool. Three stretch factors were investigated for this study: 1X1X (0% draw‐processed), 1.5X1.5X (125% draw‐processed), and 2X2X (300% draw‐processed) and applied to all three protein formulations (rHIFα, 1:1 rHIFα:rHIFɣ_(C387S)_, and rHIFɣ_(C387S)_).

### Fibre mechanical analysis

Fibres were allowed to dry overnight on the collection spools at 24°C and 16% humidity. Individual fibre samples were then mounted across a rectangular 19 mm opening on plastic film C‐shaped cards, as has been previously reported (Albertson *et al*., 2014). Tape and cyanoacrylate were used to secure the fibres to cards to prevent slipping during measuring and testing. A Motic BA310 microscope coupled with the Motic Image Plus 2.0 program (Schertz, TX, USA) was then used to measure fibre diameters at nine different points along with the 19 mm long fibre segments. A MTS Synergie 100 tensile testing instrument with a custom 10 g load cell (Transducer Techniques, Newark, CA, USA), was used to perform the uniaxial tensile test on the fibres at an extension rate of 5 mm min^−1^ and an acquisition rate of 120 Hz. Sample sizes of twenty to twenty‐five individual fibres were tested for every protein and stretch factor combination. The displacement and load data were exported to Microsoft Excel. The raw data in Microsoft Excel, along with average fibre diameter measurements, were used to calculate the ultimate tensile strength, strain, toughness, and elastic modulus for each fibre.

### Fibre structural analysis

The collected fibre samples were also probed with Fourier‐transform infrared (FTIR) spectroscopy to evaluate the compositions of secondary protein structures present. As‐spun fibres were gently scooped out of the coagulation bath and allowed to dry in a similar fashion as the stretched fibres prior to analysis. A minimum of at least 50 individual fibres was obtained from the collection spools for each unique combination of protein (rHIFα, 1:1 rHIFα:rHIFɣ_(C387S)_, and rHIFɣ_(C387S)_) and stretch factor (1X1X, 1.5X1.5X, and 2X2X). The individual fibres were then twisted together to form a multi‐fibre bundle that was used for the spectroscopic analysis. A Varian 660‐IR instrument (Agilent, Santa Clara, CA, USA) fitted with horizontal MIRacle single reflection attenuated total reflectance module (Pike Technologies, Fitchburg, WI, USA) was used to obtain the FTIR spectra. Measurements were obtained for each bundle by wrapping it upon itself, into a small coil, and then securely clamping it directly onto the crystal stage. The collection was performed with Resolution Pro software over the spectral range of 600 to 4000 cm^−1^, with 32 scans, a resolution of 4 cm^−1^, and an aperture setting of 4 cm^−1^ at 4000 cm^−1^. Background scans were collected before each bundle with the exact conditions that were used for the bundle. Spectral correction and deconvolution for secondary structure quantification was performed at the Amide I region (~1600 to 1700 cm^−1^) using OriginPro and a similar method as previously described by (Böni *et al*., 2018). The only variation to the described method was the use of Gaussian curves for peak fitting. All secondary structure peak assignments were based upon previous assignments used for the characterization of fibrous proteins utilized by (Guo *et al*., 2018) and based upon the work by (Hu *et al*., 2006).

### Statistical analysis

All values presented are provided in mean ± standard deviation format. Analyses were first performed using a two‐factor ANOVA to determine if any statistically significant differences were present. The specific significant differences were then determined with a Tukey post hoc analysis test. A *P*‐value of <0.05 was considered statistically significant.

## Funding Information

This work was funded by the United States Department of Defense (FA8075‐14‐D‐0014) administered by Serco Inc.

## Conflict of interest

The authors declare no conflicts of interest.

## Author contributions

P.E.O and D.C contributed equally to this work. P. E.O.: Methodology, Investigation, Writing. D.C.: Methodology, Investigation. B.E.B.: Methodology, Investigation, Writing. T.I.H.: Investigation, Formal Analysis, Writing, Visualization. C.W.: Investigation. H.Z.: Investigation. B.G.: Investigation. R.V.L.: Conceptualization, Writing. J.A.J.: Funding Acquisition, Project Administration, Supervision, Conceptualization, Writing.

## Supporting information

**Fig. S1**. Pairwise comparisons of mechanical properties for all stretched fibres and β‐sheet content for all fibres, including as‐spun. An asterisk indicates a statistically significant difference with a *P*‐value ≤0.05, and a blank square indicates no statistically significant difference.

**Fig. S2**. Map of pET19K expression vector.

**Fig. S3**. Fully‐assembled syringe extrusion device. (A) BD 3 ml syringe, (B) PEEK tubing to luer‐lok female adapter, (C) One‐Piece FingerTight Fitting, and (D) PEEK tubing (0.254 mm internal diameter).

**Fig. S4**. Diagram of customized extrusion method and spinning instrument.

**Table S1**. BioFlo610 protein yields from cell mass purifications. Final BioFl0610 volumes were 95 and 68 l and 19 and 13 kg for rHIFα and rHIFɣ_(C387S)_, respectively.

**Table S2**. Fermentation K12 medium.

## References

[mbt213869-bib-0001] Albertson, A.E., Teulé, F., Weber, W., Yarger, J.L., and Lewis, R.V. (2014) Effects of different post‐spin stretching conditions on the mechanical properties of synthetic spider silk fibres. J Mech Behav Biomed Mater 29: 225–234. 24113297 10.1016/j.jmbbm.2013.09.002 PMC4068612

[mbt213869-bib-0002] Böni, L.j., Sanchez‐Ferrer, A., Widmer, M., Biviano, M.d., Mezzenga, R., Windhab, E.j., *et al*. (2018) Structure and nanomechanics of dry and hydrated intermediate filament films and fibres produced from hagfish slime fibres. ACS Appl Mater Interfaces 10: 40460–40473. 30371056 10.1021/acsami.8b17166

[mbt213869-bib-0003] Bowen, C.H., Dai, B., Sargent, C.J., Bai, W., Ladiwala, P., Feng, H., *et al*. (2018) Recombinant spidroins fully replicate primary mechanical properties of natural spider silk. Biomacromol 19: 3853–3860. 10.1021/acs.biomac.8b00980 30080972

[mbt213869-bib-0004] Copeland, C.G., Bell, B.E., Christensen, C.D., and Lewis, R.V. (2015) Development of a process for the spinning of synthetic spider silk. ACS Biomater Sci Eng 1: 577–584. 27064312 10.1021/acsbiomaterials.5b00092 PMC4826064

[mbt213869-bib-0005] Ding, D., Guerette, P.A., Hoon, S., Kong, K.W., Cornvik, T., Nilsson, M., *et al*. (2014) Biomimetic production of silk‐like recombinant squid sucker ring teeth proteins. Biomacromol 15: 3278–3289. 10.1021/bm500670r 25068184

[mbt213869-bib-0006] Edlund, A.M., Jones, J.A., Lewis, R.V., and Quinn, J.C. (2018) Economic feasibility and environmental impact of synthetic spider silk production from *Escherichia coli* . New Biotechnol 42: 12–18. 10.1016/j.nbt.2017.12.006 29277712

[mbt213869-bib-0007] Fernholm, B. (1981) Thread cells from the slime glands of hagfish (*Myxinidae*). Acta Zool 62: 137–145.

[mbt213869-bib-0008] Forman, S.M., DeBernardez, E.R., Feldberg, R.S., and Swartz, R.W. (1990) Crossflow filtration for the separation of inclusion bodies from soluble proteins in recombinant *Escherichia coli* cell lysate. J Membr Sci 48: 263–279.

[mbt213869-bib-0009] Fu, J., Guerette, P.A., and Miserez, A. (2015) Self‐assembly of recombinant hagfish thread keratins amenable to a strain‐induced α‐helix to β‐sheet transition. Biomacromol 16: 2327–2339. 10.1021/acs.biomac.5b00552 26102237

[mbt213869-bib-0010] Fu, J., Guerette, P.A., Pavesi, A., Horbelt, N., Lim, C.T., Harrington, M.J., and Miserez, A. (2017) Artificial hagfish protein fibres with ultra‐high and tunable stiffness. Nanoscale 9: 12908–12915. 28832693 10.1039/c7nr02527k

[mbt213869-bib-0011] Fudge, D.S., Gardner, K.H., Forsyth, V.T., Riekel, C., and Gosline, J.M. (2003) The mechanical properties of hydrated intermediate filaments: insights from hagfish slime threads. Biophys J 85: 2015–2027. 12944314 10.1016/S0006-3495(03)74629-3 PMC1303373

[mbt213869-bib-0012] Fudge, D.S., and Gosline, J.M. (2004) Molecular design of the α–keratin composite: insights from a matrix–free model, hagfish slime threads. Proc R Soc Lond B Biol Sci 271: 291–299. 10.1098/rspb.2003.2591 PMC1691592 15058441

[mbt213869-bib-0013] Fudge, D.S., Hillis, S., Levy, N., and Gosline, J.M. (2010) Hagfish slime threads as a biomimetic model for high performance protein fibres. Bioinspir Biomim 5: 1–8. 035002. 10.1088/1748-3182/5/3/035002 20729569

[mbt213869-bib-0014] Gosline, J.M., DeMont, M.E., and Denny, M.W. (1986) The structure and properties of spider silk. Endeavour 10: 37–43.

[mbt213869-bib-0015] Guo, C., Zhang, J., Jordan, J.S., Wang, X., Henning, R.W., and Yarger, J.L. (2018) Structural comparison of various silkworm silks: an insight into the structure–property relationship. Biomacromolecules 19: 906–917. 29425447 10.1021/acs.biomac.7b01687

[mbt213869-bib-0016] Hirota, N., Mizuno, K., and Goto, Y. (1997) Cooperative alpha‐helix formation of beta‐lactoglobulin and melittin induced by hexafluoroisopropanol. Protein Sci 6: 416–421. 9041644 10.1002/pro.5560060218 PMC2143652

[mbt213869-bib-0017] Hu, X.X., Kaplan, D.L., and Cebe, P. (2006) Determining beta‐sheet crystallinity in fibrous proteins by thermal analysis and infrared spectroscopy. Macromolecules 39: 6161–6170.

[mbt213869-bib-0018] Huemmerich, D., Helsen, C.W., Quedzuweit, S., Oschmann, J., Rudolph, R., and Scheibel, T. (2004) Primary structure elements of spider dragline silks and their contribution to pProtein solubility. Biochemistry 43: 13604–13612. 15491167 10.1021/bi048983q

[mbt213869-bib-0019] Koch, E.A., Spitzer, R.H., Pithawalla, R.B., and Parry, D.A.D. (1994) An unusual intermediate filament subunit from the cytoskeletal biopolymer released extracellularly into seawater by the primitive hagfish (*Eptatretus stouti*). J Cell Sci 107: 3133–3144. 7535307 10.1242/jcs.107.11.3133

[mbt213869-bib-0020] Koch, E.A., Spitzer, R.H., Pithawalla, R.B., Castillos, F.A., and Parry, D.A.D. (1995) Hagfish biopolymer: a type I/type II homologue of epidermal keratin intermediate filaments. Int J Biol Macromol 17: 283–292. 8580093 10.1016/0141-8130(95)98156-s

[mbt213869-bib-0021] Lefèvre, T., Rousseau, M.‐E., and Pézolet, M. (2007) Protein secondary structure and orientation in silk as revealed by raman spectromicroscopy. Biophys J 92: 2885–2895. 17277183 10.1529/biophysj.106.100339 PMC1831708

[mbt213869-bib-0022] Li, B., and Sha, M. (2017) High‐density *Escherichia coli* fermentation and protein production using the Eppendorf BioFlo® 120 bioprocess control station. 6.

[mbt213869-bib-0023] Maiti, N.C., Apetri, M.M., Zagorski, M.G., Carey, P.R., and Anderson, V.E. (2004) Raman spectroscopic characterization of secondary structure in natively unfolded proteins: alpha‐synuclein. J Am Cheml Soc 12: 2399–2408. 10.1021/ja0356176 14982446

[mbt213869-bib-0024] Negishi, A., Armstrong, C.L., Kreplak, L., Rheinstadter, M.C., Lim, L.‐T., Gillis, T.E., and Fudge, D.S. (2012) The production of fibres and films from solubilized hagfish slime thread proteins. Biomacromolecules 13: 3475–3482. 23016557 10.1021/bm3011837

[mbt213869-bib-0025] Pinto, N., Yang, F.‐C., Negishi, A., Rheinstädter, M.C., Gillis, T.E., and Fudge, D.S. (2014) Self‐assembly enhances the strength of fibres made from vimentin intermediate filament proteins. Biomacromolecules 15: 574–581. 24359121 10.1021/bm401600a

[mbt213869-bib-0026] Sampath, S., Isdebski, T., Jenkins, J.E., Ayon, J.V., Henning, R.W., Orgel, J.P.R.O., *et al*. (2012) X‐ray diffraction study of nanocrystalline and amorphous structure within major and minor ampullate dragline spider silks. Soft Matter 8: 6713–6722. 23569461 10.1039/C2SM25373A PMC3617558

[mbt213869-bib-0027] Spitzer, R.H., Downing, S.W., Koch, E.A., Salo, W.L., and Saidel, L.J. (1984) Hagfish slime gland thread cells. II. Isolation and characterization of intermediate filament components associated with the thread. J Cell Biol 98: 670–677. 6537953 10.1083/jcb.98.2.670 PMC2113076

[mbt213869-bib-0028] Stauffer, S.L., Coguill, S.L., and Lewis, R.V. (1994) Comparison of physical properties of three silks from *Nephila clavipes* and *Araneus gemmoides* . J Arachnol 22: 5–11.

[mbt213869-bib-0029] Weisman, S., Haritos, V.S., Church, J.S., Huson, M.G., Mudie, S.T., Rodgers, A.J.W., *et al*. (2010) Honeybee silk: Recombinant protein production, assembly and fibre spinning. Biomaterials 31: 2695–2700. 20036419 10.1016/j.biomaterials.2009.12.021

[mbt213869-bib-0030] Xia, X.‐X., Qian, Z.‐G., Ki, C.S., Park, Y.H., Kaplan, D.L., and Lee, S.Y. (2010) Native‐sized recombinant spider silk protein produced in metabolically engineered *Escherichia coli* results in a strong fibre. Proc Natl Acad Sci USA 107: 14059–14063. 20660779 10.1073/pnas.1003366107 PMC2922564

[mbt213869-bib-0031] Yang, Y.‐X., Qian, Z.‐G., Zhong, J.‐J., and Xia, X.‐X. (2016) Hyper‐production of large proteins of spider dragline silk MaSp2 by *Escherichia coli* via synthetic biology approach. Process Biochem 51: 484–490.

