# Supplementary material for: The next generation of protein super‐fibres: robust recombinant production and recovery of hagfish intermediate filament proteins with fibre spinning and mechanical–structural characterizations
Source: Microb Biotechnol. 2021 Jun 30;14(5):1976–89. doi: 10.1111/1751-7915.13869 (PMC8449652; doi:10.1111/1751-7915.13869)
Supplement: Supplementary file 1 — Fig. S1. Pairwise comparisons of mechanical properties for all stretched fibres and β‐sheet content for all fibres, including as‐spun. An asterisk indicates a statistically significant difference with a P‐value ≤0.05, and a blank square indicates no statistically significant difference. [file MBT2-14-1976-s005.pdf]

Tensile Strength (MPa)

	rHIFα 1X1X	rHIFα 1.5X1.5X	rHIFα 2X2X	1:1 1X1X	1:1 1.5X1.5X	1:1 2X2X	rHIFγ$_{(C387S)}$ 1X1X	rHIFγ$_{(C387S)}$ 1.5X1.5X	rHIFγ$_{(C387S)}$ 2X2X
rHIFα 1X1X	—		*		*	*	*		
rHIFα 1.5X1.5X		—	*	*	*	*	*		
rHIFα 2X2X			—	*	*	*	*	*	*
1:1 1X1X				—	*	*		*	*
1:1 1.5X1.5X					—	*	*	*	*
1:1 2X2X						—	*	*	*
rHIFγ$_{(C387S)}$ 1X1X							—	*	*
rHIFγ$_{(C387S)}$ 1.5X1.5X								—	*
rHIFγ$_{(C387S)}$ 2X2X									—

Strain (mm mm^{-1})

	rHIFα 1X1X	rHIFα 1.5X1.5X	rHIFα 2X2X	1:1 1X1X	1:1 1.5X1.5X	1:1 2X2X	rHIFγ$_{(C387S)}$ 1X1X	rHIFγ$_{(C387S)}$ 1.5X1.5X	rHIFγ$_{(C387S)}$ 2X2X
rHIFα 1X1X	—		*	*	*	*	*	*	*
rHIFα 1.5X1.5X		—	*	*	*	*	*	*	*
rHIFα 2X2X			—	*		*	*	*	*
1:1 1X1X				—	*		*	*	
1:1 1.5X1.5X					—	*	*	*	*
1:1 2X2X						—	*		
rHIFγ$_{(C387S)}$ 1X1X							—	*	*
rHIFγ$_{(C387S)}$ 1.5X1.5X								—	*
rHIFγ$_{(C387S)}$ 2X2X									—

Toughness (MJ m^{-3})

	rHIFα 1X1X	rHIFα 1.5X1.5X	rHIFα 2X2X	1:1 1X1X	1:1 1.5X1.5X	1:1 2X2X	rHIFγ$_{(C387S)}$ 1X1X	rHIFγ$_{(C387S)}$ 1.5X1.5X	rHIFγ$_{(C387S)}$ 2X2X
rHIFα 1X1X	—		*	*	*		*		*
rHIFα 1.5X1.5X		—	*	*	*		*		*
rHIFα 2X2X			—			*	*	*	
1:1 1X1X				—		*	*	*	*
1:1 1.5X1.5X					—	*	*	*	
1:1 2X2X						—	*		*
rHIFγ$_{(C387S)}$ 1X1X							—	*	*
rHIFγ$_{(C387S)}$ 1.5X1.5X								—	*
rHIFγ$_{(C387S)}$ 2X2X									—

Elastic Modulus (GPa)

	rHIFα 1X1X	rHIFα 1.5X1.5X	rHIFα 2X2X	1:1 1X1X	1:1 1.5X1.5X	1:1 2X2X	rHIFγ$_{(C387S)}$ 1X1X	rHIFγ$_{(C387S)}$ 1.5X1.5X	rHIFγ$_{(C387S)}$ 2X2X
rHIFα 1X1X	—		*		*	*			
rHIFα 1.5X1.5X		—	*		*		*		
rHIFα 2X2X			—	*		*	*	*	*
1:1 1X1X				—	*	*			
1:1 1.5X1.5X					—	*	*	*	*
1:1 2X2X						—	*	*	*
rHIFγ$_{(C387S)}$ 1X1X							—		
rHIFγ$_{(C387S)}$ 1.5X1.5X								—	
rHIFγ$_{(C387S)}$ 2X2X									—

β -Sheet Content (%)

	rHIFα As-Spun	rHIFα 1X1X	rHIFα 1.5X1.5X	rHIFα 2X2X	1:1 As-Spun	1:1 1X1X	1:1 1.5X1.5X	1:1 2X2X	rHIFγ$_{(C387S)}$ As-Spun	rHIFγ$_{(C387S)}$ 1X1X	rHIFγ$_{(C387S)}$ 1.5X1.5X	rHIFγ$_{(C387S)}$ 2X2X
rHIFα As-Spun	—	*	*	*		*	*	*		*	*	*
rHIFα 1X1X		—			*		*		*			
rHIFα 1.5X1.5X			—		*				*			
rHIFα 2X2X				—	*				*			
1:1 As-Spun					—	*	*	*		*	*	*
1:1 1X1X						—	*		*			
1:1 1.5X1.5X							—		*	*		*
1:1 2X2X								—	*			
rHIFγ$_{(C387S)}$ As-Spun									—	*	*	*
rHIFγ$_{(C387S)}$ 1X1X										—		
rHIFγ$_{(C387S)}$ 1.5X1.5X											—	
rHIFγ$_{(C387S)}$ 2X2X												—
