# Supplementary figures and images for: The next generation of protein super‐fibres: robust recombinant production and recovery of hagfish intermediate filament proteins with fibre spinning and mechanical–structural characterizations
Source: Microb Biotechnol. 2021 Jun 30;14(5):1976–89. doi: 10.1111/1751-7915.13869 (PMC8449652; doi:10.1111/1751-7915.13869)

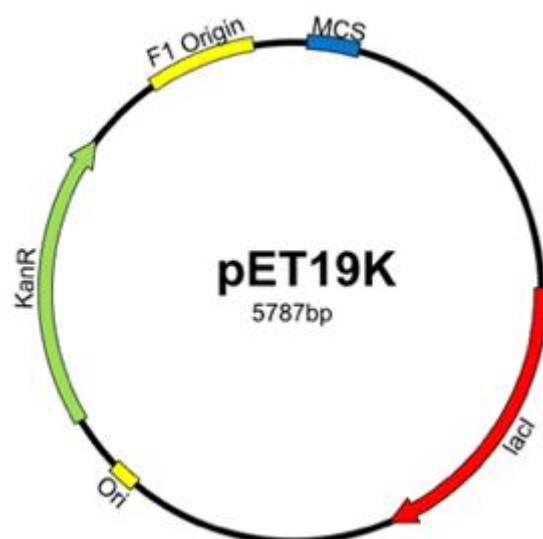

**Fig. S2.** Map of pET19K expression vector.

Supplement: Supplementary file 2 — Fig. S2. Map of pET19K expression vector. [file MBT2-14-1976-s003.pdf]

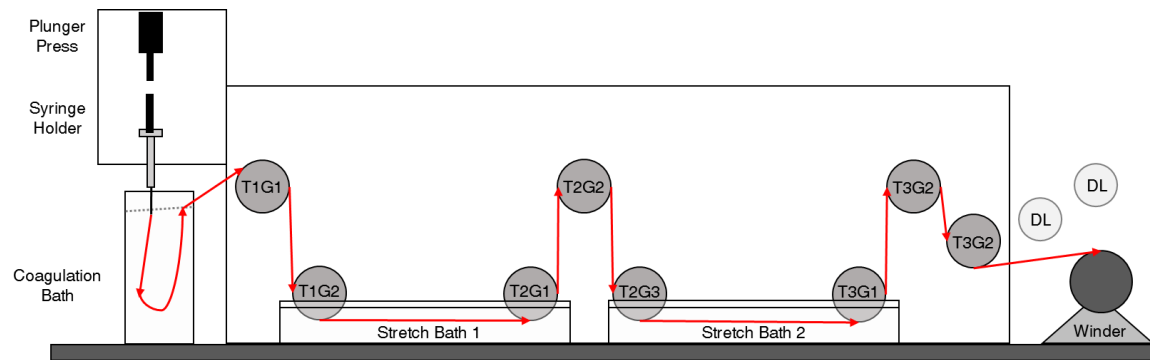

**Fig. S4.** Diagram of customized extrusion method and spinning instrument.

Supplement: Supplementary file 4 — Fig. S4. Diagram of customized extrusion method and spinning instrument. [file MBT2-14-1976-s002.pdf]
