# Supplementary material for: The next generation of protein super‐fibres: robust recombinant production and recovery of hagfish intermediate filament proteins with fibre spinning and mechanical–structural characterizations
Source: Microb Biotechnol. 2021 Jun 30;14(5):1976–89. doi: 10.1111/1751-7915.13869 (PMC8449652; doi:10.1111/1751-7915.13869)
Supplement: Supplementary file 3 — Fig. S3. Fully‐assembled syringe extrusion device. (A) BD 3 ml syringe, (B) PEEK tubing to luer‐lok female adapter, (C) One‐Piece FingerTight Fitting, and (D) PEEK tubing (0.254 mm internal diameter). [file MBT2-14-1976-s004.pdf]

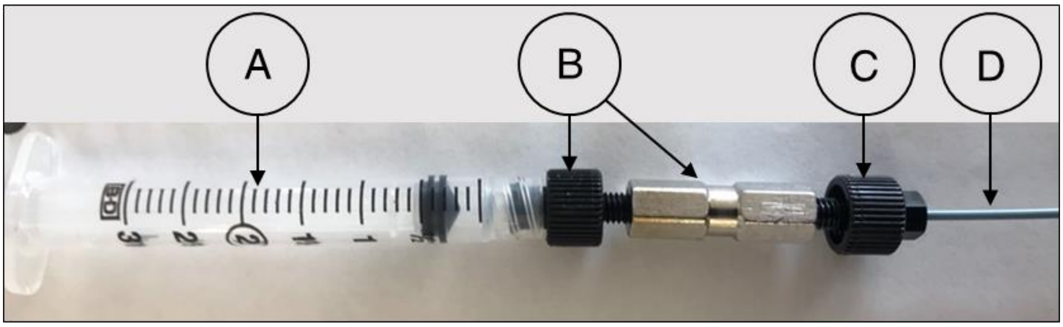

Fig. S3. Fully-assembled syringe extrusion device. (A) BD 3 mL syringe, (B) PEEK tubing to luer-lok female adapter, (C) One-Piece FingerTight Fitting, and (D) PEEK tubing (0.254 mm internal diameter).
