# Supplementary material for: The next generation of protein super‐fibres: robust recombinant production and recovery of hagfish intermediate filament proteins with fibre spinning and mechanical–structural characterizations
Source: Microb Biotechnol. 2021 Jun 30;14(5):1976–89. doi: 10.1111/1751-7915.13869 (PMC8449652; doi:10.1111/1751-7915.13869)
Supplement: Supplementary file 5 — Table S1. BioFlo610 protein yields from cell mass purifications. Final BioFl0610 volumes were 95 and 68 l and 19 and 13 kg for rHIFα and rHIFɣ(C387S), respectively. [file MBT2-14-1976-s006.pdf]

Table S1. BioFlo610 protein yields from cell mass purifications. Final BioFl0610 volumes were 95 L and 68 L and 19 kg and 13 kg for rHIFα and rHIFɣ$_{(C387S)}$, respectively.

rHIFα

Purification #	Cell Mass (g)	Volume Equivalent (L)	Recovered Protein (g)	Volumetric Yield (g L^{-1})	Mass Yield (g kg^{-1})
1	400	2	17	8.5	42.5
2	400	2	13	6.5	32.5
3	400	2	14	7	35
4	400	2	15	7.5	37.5
5	400	2	18	9	45
6	400	2	16	8	40
Average	400	2	15.5 ± 1.9	7.8 ± 0.9	38.8 ± 4.7

rHIFɣ$_{(C387S)}$

Purification #	Cell Mass (g)	Volume Equivalent (L)	Recovered Protein (g)	Volumetric Yield (g L^{-1})	Mass Yield (g kg^{-1})
1	400	2.1	15	7.1	37.5
2	400	2.1	19	9	47.5
3	400	2.1	20	9.5	50
4	400	2.1	17	8.1	42.5
5	500	2.5	23	9	46
Average	420 ± 45	2.2 ± 0.2	18.8 ±	8.5 ± 1.0	44.7 ± 4.9
