# Supplementary material for: The next generation of protein super‐fibres: robust recombinant production and recovery of hagfish intermediate filament proteins with fibre spinning and mechanical–structural characterizations
Source: Microb Biotechnol. 2021 Jun 30;14(5):1976–89. doi: 10.1111/1751-7915.13869 (PMC8449652; doi:10.1111/1751-7915.13869)
Supplement: Supplementary file 6 — Table S2. Fermentation K12 medium. [file MBT2-14-1976-s001.pdf]

Table S2. Fermentation K12 medium.

Initial Medium:	
Potassium phosphate monobasic anhydrous	2 g L^{-1}
Potassium phosphate dibasic trihydrate	4 g L^{-1}
Ammonium phosphate dibasic anhydrous	5 g L^{-1}
Yeast Extract	5 g L^{-1}
Tryptone	2.5 g L^{-1}
**Added after autoclaving of the initial medium:**	
Glucose	25 g L^{-1}
Magnesium sulfate heptahydrate	0.5 g L^{-1}
Thiamine	2.5 mg L^{-1}
X1000 trace metal solution	1 mL L^{-1}
**Glucose feeding solution:**	
Glucose	500 g L^{-1}
Tryptone	10 g L^{-1}
Magnesium sulfate heptahydrate	10 g L^{-1}
Thiamine	40 mg L^{-1}
X1000 trace metal	1 mL L^{-1}
**[X1000] trace metal solution in water:**	
Sodium chloride	25 g L^{-1}
Zinc sulfate heptahydrate	5 g L^{-1}
Manganese chloride tetrahydrate	20 g L^{-1}
Ferric chloride hexahydrate	23.8 g L^{-1}
Cupric sulfate pentahydrate	2 g L^{-1}
Boric acid	2.9 g L^{-1}
Sodium molybdate dihydrate	2.5 g L^{-1}
6 N sulfuric acid	62.5 mL L^{-1}
